# Artificial intelligence based techniques for caries risk prediction and assessment: A scoping review

**DOI:** 10.1016/j.jobcr.2025.08.027

**Published:** 2025-09-10

**Authors:** Sonal Bhatia, Vinay Kumar Gupta, Sumit Kumar, Gaurav Mishra, Seema Malhotra, Khushboo Arif, Atrey Pai Khot, Aman Rajput, Angad Mahajan

**Affiliations:** a Department of Public Health Dentistry, King George's Medical University, Lucknow, Uttar Pradesh, India; b Community Health Centre, Chinhat, Lucknow, Uttar Pradesh, India; c Department of Conservative Dentistry and Endodontics, Maulana Azad Institute of Dental Sciences, New Delhi, India

## Abstract

**Objective:**

The purpose of this scoping review was to systematically search through the evidence for the applications of artificial intelligence (AI) for caries risk assessment (CRA) or prediction (CRP), determine the scope of the methodologies used, summarize their performance metrics, and report limitations and challenges (if any).

**Design:**

A structured and comprehensive search of three electronic databases, MEDLINE, EMBASE, and Google Scholar, was performed to yield results from 2013 to 2023. Studies were selected through title, abstract, and full-text screening based on the selection criteria. Charting of the extracted data was performed using a self-designed checklist with eight dimensions.

**Results:**

The electronic database search retrieved 3059 articles. Ultimately, 13 articles were included in the review. The most used methods were logistic regression (n = 9) and random forest (n = 8). The performance of the included models was measured variably. The reported performance metrics of the models were heterogeneous in nature; the sensitivity ranged from 0.59 to 0.996, while the specificity ranged from 0.531 to 0.943. The most frequently utilized predictors include socio-demographic factors, oral hygiene habits, and dietary habits.

**Conclusion:**

Of the AI-based CRA models analyzed, machine learning algorithms were most frequently used. This review highlights that AI methods most probably show superior specificity and better performance than traditional methods. The application of these algorithms can have significant implications for the population impacted by pertinent chronic diseases that are avoidable through risk reduction, such as dental caries.

## Graphical abstract

**Figure:**
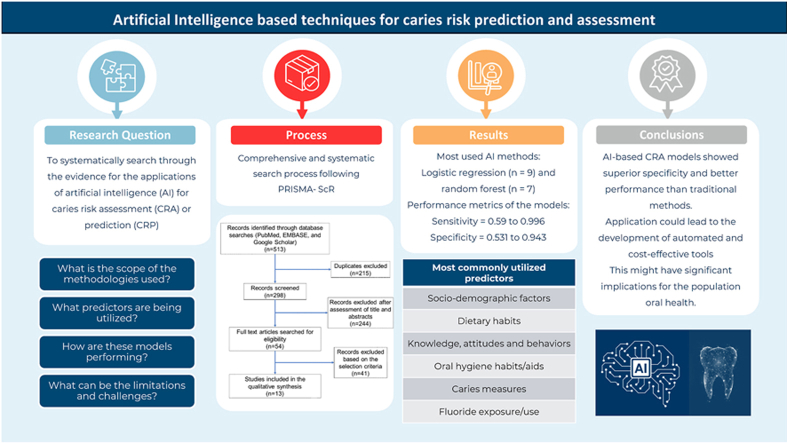

## 1 Introduction

Artificial intelligence (AI) technologies are one of the most consequential technologies and hold the power to revolutionize numerous sectors, including healthcare. AI systems are machines that mimic human intelligence and use cognitive processes to perform tasks. They achieve this by collecting and processing data, interpreting the information, applying reasoning, and deciding upon optimal actions to attain predefined objectives. These AI systems can employ numeric models or symbolic rules and can adapt their behavior by analyzing the consequences of previous actions.^1^

AI encompasses various components, such as machine learning (ML), neural networks (NNs), and deep learning (DL). ML is the process of predicting outcomes based on extensive ‘learning’ of data and interpreting it without human intervention. NNs aim to create interconnected pathways for information transmission, similar to human neurons. DL, a subset of ML, involves data analysis using different computational layers.^2^

With recent advancements in data acquisition and computational capabilities, AI has expanded across diverse domains. In healthcare, AI has been applied predominantly in the domains of diagnosis and prognosis prediction.^3^ There are a multitude of applications in dentistry that enable dentists to provide high-quality dental care. These are auxiliary tools for enhancing diagnostic accuracy, treatment planning, predicting prognosis or treatment outcomes, and managing electronic dental records.^4^

Preventive approaches in dentistry are considered time-saving and cost-effective measures for halting the initiation or progression of dental disease. AI may facilitate real-time patient data monitoring and long-term outcome assessment and enable dentists to intervene at early stages.^5^ Despite some research on AI applications in dentistry, limited information is available on their specific use for preventing dental diseases.

Caries risk assessment (CRA) is aimed at the early diagnosis of caries and the introduction of appropriate preventive measures. CRA can be performed using a wide range of software or questionnaires.^6^ Among these, the American Dental Association (ADA) Caries Risk Assessment, Caries Management By Risk Assessment (CAMBRA), American Academy of Pediatric Dentistry Caries Assessment Tool (AAPD CAT) and Cariogram are the most well-known. These consider multiple factors, including behavioral, socio-demographic, and other factors in addition to clinical factors. For example, Cariogram utilizes salivary rate and buffering capacity as well as lactobacilli and streptococci levels. Some of these need laboratory testing, which adds to the expense and time involved.^7^

ML algorithms show promise for altering the traditional methods of caries risk assessments. In ML, classification algorithms are trained using datasets. Subsequently, they can find patterns in the training data and, hence, automatically produce rules for data mining or forecasting the future behavior of variables. Due to its ability to analyze large and complex datasets, it can aid in clinical decision-making by diagnosing and predicting health conditions.^8^ This approach can help in the identification of high-risk individuals and aid in the development of targeted prevention strategies, such as personalized oral hygiene instructions, dietary counseling, and planned recall intervals.^9^ Previous reviews have elaborated on the applications of AI in caries diagnosis and prediction,^10^^,^^11^ however, have not yet summarized the use separately in the domain of caries risk assessment. Therefore, we intend to examine the applications of AI only in the use case of caries risk assessment or caries prediction. The objective of this review is to investigate this paradigm through a systematic presentation of the breadth of the available evidence. We also wish to inform practitioners, policymakers, and the health informatics community about these emerging models to support future oral health research and practice initiatives.

## 2 Methods

### 2.1 Study design

The aim was to conduct a systematic search of the published literature database on the use of AI methods for caries risk prediction. It further scopes the accuracy of prediction, range of methodologies, algorithm architectures, and key outcomes, as well as exploring the reported challenges and limitations. A scoping review approach was selected for the systematization of evidence due to the paucity of a comprehensively reviewed body of literature and the possibility of a heterogeneous data pool.^12^ A scoping review approach is more thorough than a narrative review approach because it uses systematic search functions to locate pertinent data.

A study protocol was developed *a priori*. This scoping review was developed using the Joanna Briggs Institute (JBI) modifications^13^ of the Arksey and O'Malley^14^ methodological framework to ensure rigor and facilitate reproducibility. It was conducted in accordance with the PRISMA-ScR checklist.^15^

### 2.2 Selection criteria

A framework comprising population, concept, and context (PCC) was adopted. The inclusion criteria were: full-text original articles addressing AI-based techniques for caries risk assessment or prediction written in the English language and published since 2013. This temporal restriction was applied due to the recent emergence of the concept.^16^ The exclusion criteria were: duplicate articles; unpublished literature; reviews, books or chapters; guidelines; abstracts; editorials; commentaries; or opinion articles.

#### 2.2.1 Population

Any type of population characteristics was not restricted, such as, age, gender, geography, etc. The inclusion criteria included studies pertaining to the use of AI-based algorithms for the purpose of predicting or assessing risk for dental caries in any population.

#### 2.2.2 Concept

Various procedures are used as part of current methods for CRA. These may include collecting information on certain risk factors, such as past caries experience, socio-demographics, oral hygiene habits, dietary assessments, microbial counts, and salivary properties. These are often followed up with a subjective assessment of the risk level.^6^ Risk identification can facilitate a clinician to analyze the care pathway. Clinical Pathway Analyses (CPA) require CRA as a first crucial step, which aids in instituting proper healthcare decisions. A CRA method should include a description of the various predictors and the methods implemented to obtain risk levels. Furthermore, the risk levels should be categorizable or measurable. Facilitation of this process through any AI-based algorithm is considered in this review.

#### 2.2.3 Context

To be included in the review, data collection for CRA could have been executed using traditional or advanced methods. However, the data analysis must be performed using AI-based statistical techniques to produce an output. In other words, either data processing, analysis, or inference should involve AI methods. There is no other specific context for a study to be included, keeping in mind the broad scope of the study.

### 2.3 Systematic search strategy

Three databases were comprehensively searched i.e. MEDLINE (PubMed), EMBASE (Elsevier), and Google Scholar. The search was performed by two reviewers from August to September 2023. The final search concluded on September 24, 2023.

The search strategy involved a two-stage design. In the initial stage, we used broad terms to capture titles related to "AI for dentistry" and “AI for preventive dentistry”, as preliminary scoping indicated the challenge of specifying the dimensions within the domain of "caries risk assessment”. The second stage involved trawling the abstracts for articles specifically addressing CRA. The detailed search strategy can be accessed via the supplementary tables 1, 2 and 3.

**Fig. 1:**
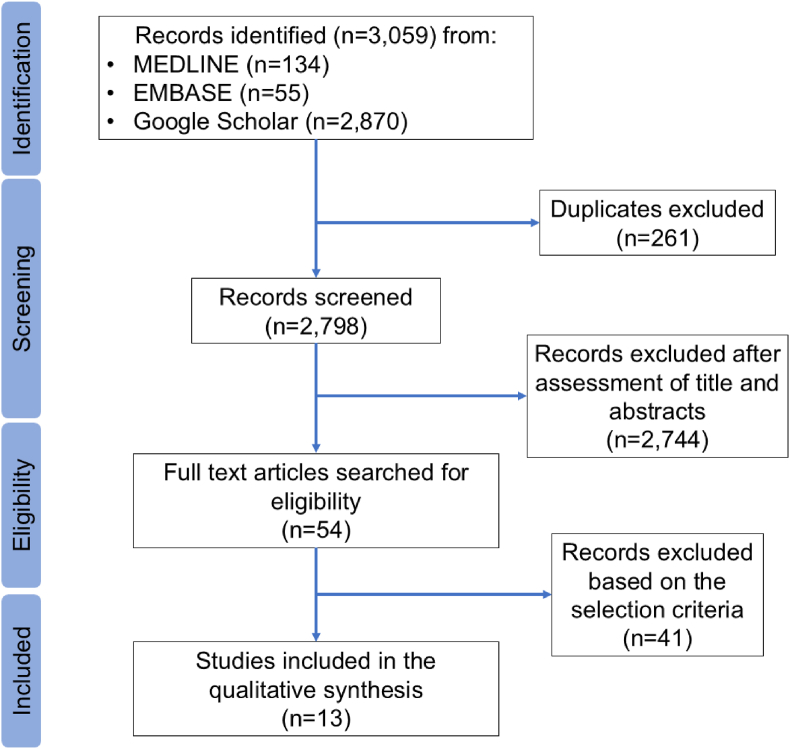
PRISMA-ScR flow diagram.

Following their extraction, the articles underwent eligibility screening using the aforementioned selection criteria. In Fig. 1, the two-phase process is shown. In the first round, titles and abstracts were separately scrutinized. All articles were retrieved and imported into the “Rayyan” software (https://www.rayyan.ai/) to perform the collaborative title and abstract screening. In the second phase, full-text papers were reviewed to determine if the study could be included in the final analysis. The selected articles were then imported to the bibliographic manager EndNote X8 (Thomson Reuters, New York) to maintain records. Wherever necessary, a third author was consulted to reach a consensus on any differences.

A self-designed datasheet was checked for its content validity through a group of experts. The items were validated on the basis of their relevance, clarity and completeness. Using this checklist, three reviewers independently extracted and documented the data. This included eight attributes: i) authors, publication year, country; ii) study design; iii) population and sample size; iv) caries risk predictors utilized; v) AI algorithm used and methodological characteristics; vi) performance of the AI method; vii) key outcomes; and viii) challenges or limitations.

The characteristics of the selected studies were tabulated. After the data were recorded, a subanalysis was conducted independently by two authors to extract additional details and evaluate the data. The data analysis process was continuously revised throughout the entire duration to optimize the results.

## 3 Results

Initial electronic database search retrieved 3059 articles (MEDLINE (n = 134), EMBASE (n = 55), Google Scholar (n = 2870)). After removing duplicates, 2798 articles were screened for titles and abstracts. The remaining 54 articles were assessed for eligibility and subjected to full-text analysis. Ultimately, 13 articles were included in the final analysis.

A table depicting the study characteristics, population, AI methods, key outcomes, and limitations is shown in Table 1.

**Table 1:** Key characteristics, methods, outcomes, and limitations of each study.

Study, Year, Country	Population (sample size)	Predictors utilized	AI method (algorithms used)	Performance	Outcomes	Limitations
Kang et al., 2023, Korea^23^	Children (22,288)	⋅ Socio-demographic (age, gender, religion, family income) ⋅ Past caries experience ⋅ Brush frequency ⋅ Floss usage ⋅ Discomfort ⋅ Smoking history ⋅ Snacking	ML (RF, LR, SVM, GBDT); DL (LSTM)	**For GBDT:** F1 Score = 93.8 % Precision = 99.8 % Recall = 88.4 % Accuracy = 95.2 % AUC = 95 %	⋅ Effective predictive models ⋅ Reduced feature sets models outperformed complete feature sets models	Limited generalizability
Sadegh-Zadeh et al., 2022, Iran^8^	Children, 0–5 years old (780; 600 with caries)	**Based on CRA by ADA** ⋅ Socioeconomic status (parents) ⋅ Fluoride exposure ⋅ Sugary foods/drinks ⋅ Regular dental visits ⋅ Special needs ⋅ Chemo- or radio-therapy ⋅ Eating disorders ⋅ Salivary flow-reducing medications ⋅ Cavitated or non-cavitated lesions ⋅ Visible plaque ⋅ Teeth extracted due to caries in past 3 years ⋅ Proximal restorations ⋅ Orthodontic appliances ⋅ Unusual tooth morphology	ML (DT, XGBoost, KNN, LR, MLP, RF, SVM)	Accuracy (both MLP and RF) = 97.4 % Precision (MLP) = 98 % Recall (MLP) = 98 %	⋅ Present caries, sugar consumption, low parental socioeconomic level, irregular dental visits, and low fluoride exposure were contributory towards high caries risk ⋅ Socio-economic factors, past caries experience and biological factors are important attributes for caries in children ⋅ Best models were MLP, RF, and SVM (kernel = ‘rbf’) ⋅ Accurate, unbiased and generalizable classifier	Limited caries-free data; Bacterial count omitted; Pilot study (limited sample size); May not be applicable to other countries
Qu et al., 2022, China^22^	Children, 12–60 months old (424)	⋅ Demographic data (children and parents) ⋅ DMF Index ⋅ Oral health–related behaviors ⋅ Parental oral health ⋅ perception ⋅ Children's general health status ⋅ Oral hygiene ⋅ Regular dental visits ⋅ Snack times, feeding pattern ⋅ Parental oral health information- seeking behaviors ⋅ Mother's childbearing age, child delivery method and pregnant nutrition	ML (RF, LR, Adaboost)	**For RF:** Accuracy = 0.82 Se = 0.76 Sp = 0.88 PPV = 0.86 NPV = 0.20 AUC = 0.91 F1 Score = 0.84 Youden's Index = 0.68 Optimal Cut-off = 0.62	⋅ RF showed the best internal validation performance ⋅ Novel CRP model: focused on dental-related behaviors ⋅ Excluded past caries experience and biological information as indicators ⋅ Unique approach to predicting caries before onset in children ⋅ Community-Level Prediction	Self-Reported Memory Bias; Lack of External Validation; Not all predictors are causative for caries
Pang et al., 2021, China^29^	Adolescents, 13–14 years old (1055)	⋅ Socio-demographic factors ⋅ Oral health-related behaviors ⋅ Past caries experience ⋅ Plaque index ⋅ Cariostat ⋅ DMFT ⋅ ICDAS ⋅ Saliva flow rate ⋅ Saliva buffering capability ⋅ Salivary pH ⋅ SNP markers	ML (RF and LR)	**For RF:** AUC = 0.78 (training) AUC = 0.73 (testing) Se; Sp (high risk) = 59 %; 68.3 % Se; Sp (low risk) = 29.4 %; 62.5 % PPV = 73.5 NPV = 52.8 Youden's Index = 0.27	⋅ “Past caries experience” was the most powerful risk predictor ⋅ First CRP model structured on both genetic and environmental factors using ML ⋅ Accurate estimation of risks (“high” and “very high” caries risk individuals) ⋅ High discrimination ability	Underestimation of caries risk for “low” and “very low” risk individuals
Karhade et al., 2021, USA^17^	Children, 3–5 years old (6404)	**For training:** ⋅ Socio- demographic data ⋅ Parent and child-reported oral health status ⋅ Parental education ⋅ ICDAS ⋅ Behavioral data ⋅ Fluoride exposure ⋅ Diet/nutrition ⋅ Dental home ⋅ Oral hygiene **Inputs for classifier:** ⋅ Child's age ⋅ Parent-reported child oral health status	ML using Google Cloud AutoML (DT)	**Best model with 2 predictors:** AUC = 0.74 Se = 0.67 PPV = 0.64	⋅ Best model used child's age + parent-reported child oral health status ⋅ Modest performance ⋅ External validation performed using the NHANES survey	Subjective nature of parental perceptions; Intended to use as a screening tool more than CRA
Wu et al., 2021, USA^20^	Mother-children dyads (37)	⋅ Socio-demographic data (age, race, ethnicity) ⋅ Tooth brushing frequency ⋅ Daycare attendance ⋅ Inhaler use ⋅ Plaque index ⋅ Oral Candida status ⋅ 16s rRNA sequencing	ML (LASSO-Penalized LR)	**AUC:** Child saliva model = 0.82 Child plaque model = 0.78 Mother plaque model = 0.73	⋅ Novel multifactorial model using socio-demographic, medical, and dental characteristics with oral hygiene habits, daycare attendance, oral Candida status, and 16s relative species abundance. ⋅ Both cariogenic and protective bacteria utilized	Limited sample size; Limited generalizability; Model not based on longitudinal onset of caries
Zaorska et al., 2021, Poland^21^	Children, 20–40 months old (262)	⋅ Demographic data ⋅ Genotyping of SNPs	NN and LR	**NN:** Prediction accuracy = 90.9 %–98.4 % **LR:** Accuracy = 93 % Se = 89.6 % Sp = 95.7 %	⋅ Novel genetic caries prediction model ⋅ Most important predictors were AMELX and TUFT1 ⋅ High caries prediction rates	Need for larger sample and validation of the model
Park et al., 2021, Korea^18^	Children, 1–5 years old (4195)	⋅ Socio-demographic data (age, sex, siblings, and income level) ⋅ Oral hygiene practices (toothbrushing frequency) ⋅ Maternal attributes (education level, birthing age, floss or interdental brush usage, and brushing frequency)	ML (XGBoost, RF and Light GBM LR)	**For LR (final model):** AUC = 0.784 Se = 0.799 Sp = 0.531	⋅ Models could accurately predict ECC with identification of high-risk groups ⋅ ML and LR models had almost similar predictive performance	Skewed data distribution; Clinical examination reliance; Missing variables; Causality limitation
Grier et al., 2021, USA^24^	Children, 1–3 years old, eligible for medical insurance (56)	⋅ Socio-demographic data ⋅ Clinical information ⋅ Oral microbiota composition ⋅ Time-dependent data ⋅ Caries status ⋅ Individual Taxa abundance	ML (RF, Gradient Tree)	AUC = 0.89 Se = 0.875 Sp = 0.85 Accuracy = 85.5 %	Oral microbiota markers can be accurate predictors in caries-free children	Sample size; Difficult to establish causality
Ramos-Gomez et al., 2021, USA^25^	Children aged 2–7 years (Questionnaire administered to parents) (182)	⋅ Socio-demographic data ⋅ Fluoridated water and toothpaste use ⋅ Self-reported oral health status ⋅ Physical status of teeth and gingiva ⋅ Dental esthetics ⋅ Access to dental services (including preventive treatments) ⋅ General wellbeing ⋅ Parental preventive actions ⋅ Oral hygiene habits	ML (RF)	**For testing set:** i) Active caries model Accuracy = 0.62 Sensitivity = 0.57 Specificity = 0.63 ii) Caries experience model Accuracy = 0.73 Sensitivity = 0.92 Specificity = 0.55	Dental appearance and aesthetics were associated with oral hygiene and are important predictors of dental caries	Small sample size; Low percentage of active caries; Reliance on parent-reported outcomes; Selection bias; Lack of patient-reported outcomes
Liu et al., 2020, China^26^	Geriatric population 65–74 years old (1144)	⋅ Socio-demographic data ⋅ Eating pattern ⋅ Oral health-related behavior ⋅ Oral health knowledge ⋅ Oral hygiene habits ⋅ Fluoride toothpaste use ⋅ Medical treatment ⋅ Frequency of eating sweet foods ⋅ Smoking/drinking habit	ANN (LR and GRNN)	AUC (LR) = 0.578 AUC (GRNN) = 0.777 Se (LR) = 84.52 % Se (GRNN) = 85.16 % Sp (LR) = 31.08 % Sp (GRNN) = 70.27 % Optimal cut-off (LR) = 0.606 Optimal cut-off (GRNN) = 0.680 Youden's index (LR) = 0.370 Youden's index (GRNN) = 0.638	Primary risk factors associated with dental caries in elderly: toothache history, smoking/drinking, fluoride toothpaste, residence type, sugar consumption and toothbrushing frequency	Difficult to interpret results and judge contribution of variables with GRNN; Predictors might not be accurate as it was not a cohort study
Udod et al., 2020, Ukraine^50^	Subjects aged 6–7, 12–15 and 35–44 years (73)	⋅ Cfc and cfcex (extracted teeth) ⋅ OHI-S ⋅ SFARE according to TER	“CariePro” software based on ANN	Prediction accuracy = 83.56 %	⋅ “CariesPro” solves disadvantages of “Cariogram” i.e. it gives the no. of possible carious lesions in a particular patient over time ⋅ Facilitates timely and individualized measures of caries prevention	–
Hung et al., 2019, USA^19^	Adults (5135)	⋅ Socio-demographic data (education, income) ⋅ Last dental visit ⋅ TV watch hours ⋅ Computer use hours ⋅ Self-perceived oral health status ⋅ Alcohol use ⋅ Sunscreen use ⋅ Oral hygiene aids ⋅ Cholesterol medication	ML (SVM, XGBoost, RF, KNN and LR)	**Best model with 15 predictors (SVM):** Accuracy = 97.1 % Precision = 95.1 % Se = 99.6 % Sp = 94.3 % AUC = 0.997	⋅ Most critical indicators: age, income, last dental visit and TV watch hours. ⋅ Sunscreen use, computer use and cholesterol medicine were unique indicators	Newer set of variables utilized; Focus on discovery of new variables; Covariances were not considered

∗Se: Sensitivity, Sp: Specificity, AUC: Area under the curve, PPV: Positive Predictive value, NPV: Negative Predictive Value.

### 3.1 Study design

The populations studied covered diverse age groups, from children aged 0–5 years to older adults aged 65–74 years. However, most studies focused on children (n = 9). The sample sizes of the studies varied from a minimum of 37 to a maximum of 22,288. The majority of the studies employed a longitudinal design (n = 10), while others were cross-sectional in nature (n = 4). The data were collected from surveys, questionnaires, and clinical examinations. Three studies obtained data from nationwide surveys.^17^, ^18^, ^19^ The data were also obtained through general practice and hospital records.

### 3.2 Key predictors

Various predictors have been utilized across studies. This information has been tabulated in Table 2. The most widely used predictor was socio-demographics (n = 12), followed by caries measures (n = 7) and oral hygiene habits (n = 7). Certain novel predictors were used in isolation in two papers, such as TV and computer use hours, sunscreen use, cholesterol medication, daycare attendance^19^; and inhaler use.^20^ Several studies incorporated genetic markers (e.g., SNPs) and oral microbiota composition as predictors. For example, Zaorska et al. (2021)^21^ achieved an accuracy of 90.9–98.4 % using neural networks to predict caries based on genetic data. Studies like Qu et al. (2022),^22^ developed community-level predictive models, emphasizing dental-related behaviors and parental influence on early childhood caries.

**Table 2:** Distribution of predictors utilized in the various studies.

Predictors used	No. of studies (n)	Examples
Socio-demographic-economic factors	12	Age, sex, family income, education level, race, ethnicity, religion
Oral hygiene habits/aids	7	Toothbrushing frequency, floss and interdental brush usage
Caries measures	7	DMFT, ICDAS, past carious experience, presence of caries/cavitation
Oral health knowledge, attitudes, and behaviors	4	Dental health related behaviors, dental health knowledge, dental health perception
Dietary habits	4	Consumption of sugar-sweetened beverages and snacks, frequency of eating sweet foods, eating pattern, snacking
Fluoride exposure/use	4	Use of fluoridated tap water, fluoridated toothpaste
Perceived/reported oral health status	3	Self-reported or parent-reported oral health status
Plaque measures	3	Plaque index, visible plaque
Microbiota measures	3	Cariostat, oral microbiota composition, oral Candida status, individual and relative taxa abundance of plaque and salivary microorganisms
Utilization of dental services	3	Dental home, Regular dental visits, Last dental visit
Adverse habits	3	Smoking history, Drinking alcohol
Parental/Familial factors	2	Parental education, income, oral hygiene, birthing age, health preventive actions taken for children, family dental caries status
Medications	2	Medications reducing salivary flow, inhaler use, cholesterol medication
Genetic markers	2	SNP markers
Salivary measures	1	Saliva flow rate, saliva buffering capability, salivary pH
Dental treatment history	1	Extractions due to caries (past 36 months), proximal restorations, orthodontic appliances
Miscellaneous	–	TV watch hours, computer use hours, sunscreen use, inhaler use, cholesterol medicine use, daycare attendance, unusual tooth morphology

### 3.3 AI methods

Table 3 shows how often the AI methods were used in separate studies. Total uses, in this context, signify whether the algorithm was used alone or in combination with others and do not represent a unique use case for each study. ML (n = 11), NN (n = 3), and DL (n = 1) were the three main AI methods. Various ML methods were utilized, including random forests (RF), logistic regression (LR), support vector machines (SVM), gradient boosting decision trees (GBDT), and deep learning (DL) algorithms like LSTM. Additionally, novel tools such as Google Cloud AutoML were also explored.

**Table 3:** Frequency of use of each AI method in the selected studies.

Method used	Total uses	Method used	Total uses
RF	8	MLP	1
Decision Tree	2	LR^b^	9
SVM	3	Neural Networks	3
Gradient boosting^a^	7	LSTM (DL)	1
KNN	2		

a includes (XGBoost, Adaboost, GBDT, Light GBM).

b includes LASSO-penalized LR.

**Fig. 2:**
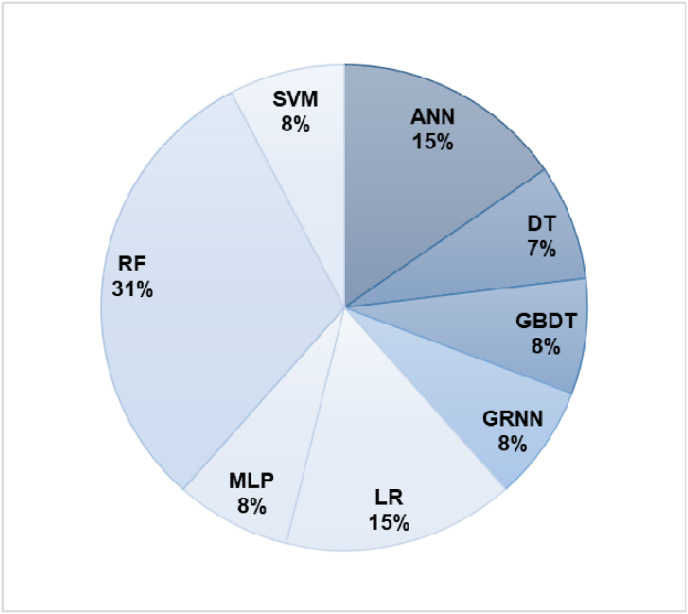
Frequency of the best-performing algorithms across various studies.

Fig. 2 provides a depiction of the best-performing algorithms reported across the studies, not considering their frequency of use. These counts are not representative of their unique use cases, i.e., they may be used in varying combinations with other algorithms.

### 3.4 Performance metrics

**Fig. 3:**
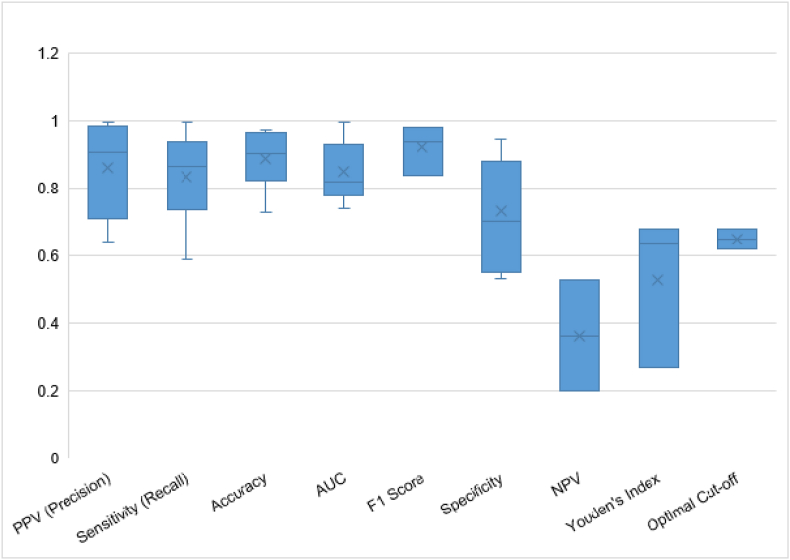
Boxplot of the performance measures: accuracy (n = 8), AUC (n = 9), F1 score (n = 3), NPV (n = 2), PPV (n = 6), sensitivity (n = 10), specificity (n = 7), Youden's index (n = 3), and optimal cutoff (n = 2) reported by selected studies.

All the studies have reported several performance metrics for prediction which were heterogeneous and variable in nature. Hence, a boxplot of the performance metrics and their frequency of use is shown in Fig. 3. If a study used multiple methods, the performance metric for the best-performing method was included. Metrics for the testing set were included over those for the training set, wherever applicable. Moreover, measures for predicting high caries levels were included. However, it can be concluded that most studies reported high predictive performance using ML methods. The following measures and their ranges were found: sensitivity or recall (0.59–0.996); precision or PPV (0.64–0.998); NPV (0.2–0.52); specificity (0.531–0.943); AUC (0.578–0.997); and accuracy (0.62–0.974). Sensitivity and specificity were generally high in well-designed models.^8^^,^^19^ Kang et al. (2023)^23^ achieved an accuracy of 95.2 % and an AUC of 95 % using GBDT for caries prediction in children. Sadegh-Zadeh et al. (2022)^8^ reported an accuracy of 97.4 % with MLP and RF models for identifying high caries risk among children aged 0–5 years. Hung et al. (2019)^19^ demonstrated exceptional performance with SVM, achieving an AUC of 0.997 for adult caries prediction.

### 3.5 Limitations

Many studies noted challenges such as small sample sizes,^8^^,^^20^^,^^24^^,^^25^ limited external validation,^21^^,^^22^ and subjective nature of self-reported data.^17^^,^^19^^,^^22^^,^^25^ The lack of longitudinal data in some cross-sectional studies^19^^,^^23^^,^^25^^,^^26^ hindered the assessment of causality between predictors and outcomes.

## 4 Discussion

To the best of our knowledge, this is the first attempt to formally investigate the applications of AI techniques for caries risk assessment and to offer discrete evidence for their effectiveness in predicting caries. All the included studies were published from 2019 to 2023, indicating that this is a contemporary topic of research. However, insufficient data were available to verify whether the inclusion criteria had inadvertently excluded earlier studies.

### 4.1 Predictors and risk factors

The reviewed AI models had successfully identified several caries risk factors. These include socio-demographic factors, oral health-related behaviors, oral hygiene habits, biological predictors (genetic, microbial, and salivary), parental influences, and other contributory variables. The most widely used were socio-demographic data and oral hygiene habits, which are similar to the findings of a systematic review of CRA methods.^6^ Dietary habits, fluoride exposure, and oral health knowledge or behaviors were also commonly utilized. In a systematic review of CRA methods,^6^ socio-demographic variables were also included in several multivariate models, however had limited accuracy in prediction. They also concluded that dietary habits as a predictor had limited predictive value and there was insufficient evidence for oral hygiene/fluoride exposure as predictors.^6^ Although, it is already well known that these factors have crucial role to play in caries processes; improper dietary habits, such as frequent snacking and excessive sugar intake, are causative in nature, while oral hygiene and fluoride exposure act as a protective factor.^27^

Oral health surveys were key data collection tools used across studies. These offer cost-effective methods for collecting data without the need for additional laboratory testing. Three studies used data from national-level surveys for training their models.^17^, ^18^, ^19^ Ramos-Gomez et al.^25^ used questionnaire items that were predictors among children, wherein dental appearance was a strong predictor. Unmet needs and demographic factors were also significant predictors. However, relying on questionnaires, especially self-reported questionnaires, can lead to various biases.^28^

“Past caries experience” was utilized as a predictor by three studies.^8^^,^^23^^,^^29^ Numerous authors have consistently argued that "past caries" serves as the most powerful and universal risk factor for caries.^6^^,^^30^^,^^31^ Past caries experience was the strongest predictor in the Pang et al. study.^29^ However, it is a mere record of the past caries activity instead of present metabolic activity.^6^ Another effective indicator in children was the incidence of caries. One model^22^ excluded past caries experience and biological information as predictors, resulting in the loss of certain vital information while still maintaining decent adaptability. Additional factors were smoking, regular dental visits, parental education, and socioeconomic status. For the geriatric population, smoking and alcohol consumption, residence, toothache history, removable dental prostheses, and self-oral hygiene assessment were the primary risk factors,^26^ which is consistent with previous research.^32^

Additional biological data can improve the performance of CRA models. One study^24^ revealed persistent microbial markers of caries, indicating that these could be precise predictive indicators, especially for early childhood caries (ECC). In this study, Streptococcus species, R. mucilaginosa, and V. parvula were consistently important. Previously, *S. mutans* culture alone outperformed AAPD-CAT, demonstrating the utility of biological data.^33^ Pang et al.^29^ employed “cariostat” score rather than "bacterial counts" used in the Cariogram.^7^ Cariostat can assess the cariogenic potential of dental plaque using a colorimetric assay to assess the bacterial acidogenicity.

Both environmental and genetic factors have impacts on the onset and progression of caries. Hence, it is logical to consider their interactions. Two studies^21^^,^^29^ used single nucleotide polymorphisms (SNPs) as predictors. Since caries is a polygenetic disease, a single SNP cannot reliably predict it.^34^ The most important SNPs were AMELX,^21^ AQP5,^29^ and TUFT1.^21^^,^^29^ Previously, a systematic review^35^ had identified an association between SNPs and caries experience, potentially validating its use as a predictor for AI based models as well.

Some unique risk factors were also discovered, such as TV watch hours, sunscreen use, computer use, and cholesterol medicine.^19^ It is important to note that the prediction models based on AI tend to be “black box” reasoning machines. They draw conclusions without any supporting evidence,^36^ and can therefore incorporate irrelevant variables for computations. This makes it challenging to assess the contribution of variables and apply the results. Hence, the validity of such hypothetical predictors should be analyzed through further studies and should be interpreted with caution and sound professional knowledge.

In addition to identifying risk factors, AI algorithms can process a wide range of factors to arrive at judgements. Generally, multivariate models are considered superior to their single predictor counterparts.^37^ The performance of AI is generalizable due to its unbiased nature. Instead of analyzing all the data, training and test datasets could be employed. Using all available data to construct a predictive model will almost certainly result in bias in modeling, which is referred to as “overfitting”. Hence, further research is mandated to identify specific risk factors.

However, caries etiology is complex, and no single predictor can accurately define real-world situations. In conclusion, a model that takes into account the most significant factors is the most appropriate.

### 4.2 AI algorithms

ML techniques "learn" from past data to identify trends and forecast future data. Hence, more precise predictions can be obtained as the training data expands. Several supervised ML methods were employed in the studies, including logistic regression (LR), decision tree, random forest (RF), extreme gradient boosting (XGBoost), support vector machine (SVM), and K-nearest neighbors (KNN) to identify significant predictors of caries risk. Previously, ML has been successfully applied for oral cancer^38^ to predict patient survival. They also have the advantages of tolerance to overfitting, accurate modeling of nonlinear connections, and ease of implementation in medical applications.^39^

LR is typically utilized in traditional research to forecast a dichotomous variable (i.e., a maximum of two classes).^40^ One advantage of LR is that complex software is not required. However, confounding may arise when examining certain environmental, behavioral, and biological aspects. This approach should be avoided in LR because it generates overfitting of the data and spurious results.^26^

Sophisticated ML techniques consider the unequal strength of factors, ensuring that a strong factor does not overpower a weak factor, thus improving predictive power. RF uses an ensemble approach rather than a single decision tree and, hence, can reduce overfitting by averaging the results. It can also analyze nonlinear and high-dimensional datasets. RF has previously shown higher AUCs (0.944) than LR (0.708) for diabetes prediction.^41^ In our review, RF outperformed LR in various performance metrics.^8^^,^^19^^,^^22^^,^^23^^,^^29^ “Feature selection” was used in one study,^23^ wherein models trained on certain selective features outperformed their counterparts trained on complete feature sets. This approach selects the most important attributes and decreases the dataset dimensionality.

NNs are systems of interconnected nodes relaying information. Each connection holds a weight, determining the strength of association. This technique enables the detection of complicated nonlinear correlations between and within variables and achieves minimum error. In one study,^26^ GRNN model was found to be more accurate than the traditional multivariate LR model, but it was less explanatory because it failed to judge the contribution of each variable.

Although RF was reported to be the best-performing algorithm in most of the studies, it became evident that this was unclear. All of these approaches are valid methods for using AI to predict caries, even if they all operate differently.

### 4.3 Performance of AI-based CRA models

It has been reported that existing CRA methods are reliable at identifying individuals with a low caries risk but possess low accuracy.^42^ Previous studies show that the AAPD tool has a high (100 %) sensitivity and very low specificity (3 %).^33^ CAMBRA tool has a high sensitivity (94 %) and moderate specificity (44 %).^43^ Cariogram has shown a balanced performance with moderate sensitivity (66.67 %) and specificity (60 %), with an overall diagnostic accuracy of 63.33 %.^44^ There is a clear need for improved specificity to reduce false positives.

Among the reviewed studies, a variety of performance metrics were found, rendering them incomparable under the scope of this review. Despite this, it is possible to analyze the ranges. Sensitivities ranged from 0.59 to 0.996, which is consistent with those reported from traditional methods. One model^24^ could predict ECC risk up to the 2-year period prior to onset without losing sensitivity.

The specificities of the AI-based models were relatively higher (0.531–0.943) than traditional methods. Hence, AI can improve caries risk prediction with reduced false positives. This is due to their high discrimination ability.^45^ An ideal prediction model would accurately differentiate between those at risk and those without risk, which is called “discrimination”.^46^ This is often evaluated using the receiver operating characteristic (ROC) curve. An area under the curve (AUC) value < 0.6 is indicative of weak discrimination, whereas a value > 0.7 suggests a great discrimination capacity.^47^ AUC of all the reviewed models was >0.74, indicating good-excellent quality.

### 4.4 Challenges and ethical considerations

While AI algorithms have higher accuracy and validity than traditional models, the use of AI in caries risk prediction raises several ethical concerns. These include data privacy, potential biases in AI algorithms, and the need for transparency in decision-making processes.^48^ The “black box” nature renders their use worrisome i.e. the lack of clarity in how AI arrives at predictions can complicate accountability in clinical practice, emphasizing the importance of regulatory oversight to maintain ethical standards. This inherent tendency obscures interpretation and hinders its independent validity and real-world authenticity.^49^ Ensuring patient consent and safeguarding sensitive patient data are critical, as is addressing the risk of AI models perpetuating existing healthcare inequalities. AI has a probability for exhibiting bias, such as socio-economic or demographic biases, especially when AI is trained on non-diverse datasets, which can lead to discriminatory outcomes. To prevent bias, AI systems must be trained on diverse datasets and evaluated for impartiality before clinical deployment. Additionally, the lack of clear regulations and guidelines for the ethical use of AI complicates responsible implementation in healthcare.^48^ Proper training of healthcare professionals in AI use, understanding system results, and mitigating risks is essential.

It is evident that, for now, the use of AI has some ethical and humanitarian limitations. Till now, health-related decisions have been made solely by humans. Substituting AI to make clinical decisions has the real possibility of making mistakes. Even a single inaccurate decision in a clinical setting can have massive implications. Hence, AI prediction models underpin issues of accountability, transparency, and privacy.

### 4.5 Implications

AI can cause a shift in the paradigm of dental care through the transformation of the dental informatics landscape. It paves the way for the development of real-time decision-support systems aiding dental diagnosis, prognosis, and prevention. AI-powered CRA models may serve as screening tools for dental offices, governmental and nongovernmental organizations, and other settings to identify high-risk individuals. The challenges of relying solely on clinical judgement can be overcome. These could also help nondental professionals classify high-risk patients and refer them to dental professionals for early intervention. In cases where clinical data is unavailable, AI can use proxy data to impute caries status, thus enabling large-scale surveillance.^17^

AI-driven predictive modeling can facilitate efficient planning and resource allocation by accurately estimating future healthcare demands based on existing data. Hence, their use in dental screening, program evaluation, and policy planning can be foreseen. These precision dental tools could significantly impact the treatment of not only caries but other dental diseases as well. On an individual level, personalized disease risk assessments with tailored treatment plans can become possible.

While AI can effectively leverage available data and occasionally replace traditional datasets, it is not yet ready to substitute clinical examinations.^17^ Additional data and further testing in diverse populations are needed to create robust models.

### 4.6 Limitations of this review

One of the main limitations of this scoping review is that the included studies offer varied methodologies and characteristic variations. There was a lot of age variation, with many studies not defining age groups. Considering the age-specific nature of caries risk factors, it is important to segregate and draw interpretations separately. This was not in confines of the objectives of the study and can be taken up in further reviews. There was significant heterogeneity in both, the type and number of predictors which were utilized in the included studies. Hence, the outcomes of the study cannot be compared with utmost accuracy. The results could have been influenced by the predictors. Due to limitations of the scoping review design, measures to address this heterogeneity could not be employed. Another limitation was not including any grey literature. Inclusion of three databases was done in an effort to mitigate this shortcoming.

## 5 Conclusion

For caries risk assessment, a wide array of AI algorithms has been employed. They utilized socio-economic-demographic data, past caries experience, baseline caries prevalence, and other etiological factors, vastly similar to traditional CRA models. These models had good accuracy, with sensitivities ranging from 59 to 99.6 % and specificities ranging from 53.1 to 94.3 %, outperforming traditional methods. These approaches can initiate early diagnosis of caries, accurately predict future onset and hence, can aid in instating personalized caries prevention and treatment plans. Therefore, these have a huge potential in the fulfillment of fundamental goals of preventive dentistry. However, the real-world actuation of AI in caries risk prediction cannot be executed without addressing its potential ethical and humanitarian concerns.

## Patient's/guardian's consent

Not applicable.

## Author contributions

**Sonal Bhatia:** Conceptualization, Methodology, Writing- Original draft preparation. **Vinay Kumar Gupta:** Methodology, Supervision, Project administration. **Sumit Kumar:** Methodology, Writing- Reviewing and Editing. **Gaurav Mishra:** Data curation, Writing- Reviewing and Editing. **Seema Malhotra:** Writing - Review & Editing. **Khushboo Arif:** Data curation, Methodology. **Atrey Pai Khot:** Data curation, Writing- Reviewing and Editing. **Aman Rajput:** Data curation, Software. **Angad Mahajan:** Software, Visualization.

## Ethical clearance

Not applicable.

## Funding sources

This research did not receive any specific grant from funding agencies in the public, commercial, or not-for-profit sectors.

## Declaration of competing interest

The authors declare that they have no known competing financial interests or personal relationships that could have appeared to influence the work reported in this paper.

## Figures and Tables

**Table:** 

**CRP**	Caries Risk Prediction
**ECC**	Early Childhood Caries
**Se**	Sensitivity
**Sp**	Specificity
**ANN**	Artificial Neural Network
**ROC**	Receiver Operating Characteristic Curve
**AUC**	Area Under the Curve
**DMF(T)**	Decayed, Missing, Filled Teeth
**DT**	Decision Trees
**XGBoost**	Extreme Gradient Boosting
**GBM**	Gradient Boosting Machine
**GBDT**	Gradient Boosting Decision Tree
**AdaBoost**	Adaptive Boosting
**KNN**	K-Nearest Neighbors
**LASSO**	Least Absolute Shrinkage and Selection Operator
**LR**	Logistic Regression
**LSTM**	Long Short-Term Memory
**GRNN**	Generalized Regression Neural Network
**ML**	Machine Learning
**MLP**	Multilayer Perception
**NPV**	Negative Predictive Value
**PPV**	Positive Predictive Value
**RF**	Random Forest
**SVM**	Support Vector Machine
**cfc**	caries, filling, cavity
**OHI-S**	Oral Hygiene Index- Simplified
**SFARE**	Structurally-functional acid resistance of tooth enamel
**TER**	Test of Enamel Resistance
