# Supplementary material for: Artificial intelligence based techniques for caries risk prediction and assessment: A scoping review
Source: J Oral Biol Craniofac Res. 2025 Sep 10;15(6):1497–507. doi: 10.1016/j.jobcr.2025.08.027 (PMC12455132; doi:10.1016/j.jobcr.2025.08.027)
Supplement: Multimedia component 1 [file mmc1.docx]

**Supplementary Material**

**Supplementary Table 1. Search Strategy for PubMed**

|  | **Search Terms** | **Hits** |
| --- | --- | --- |
| 1 | ("Artificial Intelligence"[Mesh] OR "neural network" OR "deep learning" OR "machine learning") AND "Diet" AND "Dental Caries"[Mesh] | 1 |
| 2 | ("Artificial Intelligence"[Mesh] OR “neural network” OR “deep learning” OR “machine learning”) AND ("Dental Caries"[Mesh] AND “prediction”) | 23 |
| 3 | ("Artificial Intelligence"[Mesh] OR “neural network” OR “deep learning” OR “machine learning”) AND caries risk assessment | 21 |
| 4 | ("Artificial Intelligence"[Mesh] OR "neural network" OR "deep learning" OR "machine learning") AND Caries Prediction | 66 |
| 5 | ("Artificial Intelligence"[Mesh] OR "neural network" OR "deep learning" OR "machine learning") AND Caries risk predicting models | 23 |
|  | **TOTAL** | **134** |

**Supplementary Table 2. Search Strategy for EMBASE**

|  | **Search Terms** | **Hits** |
| --- | --- | --- |
| 1 | ('artificial intelligence'/exp OR 'neural network' OR 'deep learning' OR 'machine learning') AND 'diet'/exp AND 'dental caries'/exp AND [2013-2023]/py | 1 |
| 2 | ('artificial intelligence'/exp OR 'neural network' OR 'deep learning' OR 'machine learning') AND ('dental caries'/exp AND ('prediction and forecasting'/exp)) AND [2013-2023]/py | 41 |
| 3 | ('artificial intelligence'/exp OR 'neural network' OR 'deep learning' OR 'machine learning') AND 'caries risk assessment' AND [2013-2023]/py | 4 |
| 4 | ('artificial intelligence'/exp OR 'neural network' OR 'deep learning' OR 'machine learning') AND 'caries prediction' AND [2013-2023]/py | 9 |
| 5 | ('artificial intelligence'/exp OR 'neural network' OR 'deep learning' OR 'machine learning') AND 'caries risk predicting models' AND [2013-2023]/py | 0 |
|  | **TOTAL** | **55** |

**Supplementary Table 3. Search Strategy for Google Scholar**

| **Search Terms** | **Hits** |
| --- | --- |
| (("artificial intelligence" OR "neural network" OR "deep learning" OR "machine learning") AND ((("dental caries" AND “prediction”) OR "caries risk assessment" OR "caries prediction"))) | 2,870 |
